# Burden of microbial pathogens–associated infectious diseases in Asian older adults: a systematic analysis derived from the global burden of disease 2021

**DOI:** 10.3389/fpubh.2025.1648877

**Published:** 2025-10-07

**Authors:** Lin Chen, Yuhang Lou, Jie Yang, Kai Zhang, Xiaoli Liu, Yushi Fan, Xinyun Zhang, Qing Wang, Qianqian Yang, Qitao Chen, Meihua Wang, Ya Li, Minfeng Tong, Binbin Ren, Zhongheng Zhang, Gensheng Zhang

**Affiliations:** ^1^Department of Neurosurgery Intensive Care Unit, Department of Neurosurgery, Affiliated Jinhua Hospital, Zhejiang University School of Medicine, Hangzhou, China; ^2^Department of Critical Care Medicine, Second Affiliated Hospital, Zhejiang University School of Medicine, Hangzhou, China; ^3^Department of Emergency Medicine, Sir Run Run Shaw Hospital, Zhejiang University School of Medicine, Hangzhou, China; ^4^Center for Artificial Intelligence in Medicine, The General Hospital of PLA, Beijing, China; ^5^Department of Internal Medicine St. John’s Episcopal Hospital Far Rockaway, Far Rockaway, NY, United States; ^6^Department of Neurosurgical Intensive Care Unit, Huashan Hospital Affiliated to Fudan University, Shanghai, China; ^7^College of Mathematical Medicine, Zhejiang Normal University, Jinhua, Zhejiang, China; ^8^Department of Neurosurgery, Affiliated Jinhua Hospital, Zhejiang University School of Medicine, Jinhua, Zhejiang, China; ^9^Department of Infectious Diseases, Affiliated Jinhua Hospital, Zhejiang University School of Medicine, Jinhua, Zhejiang, China; ^10^Department of Critical Care Medicine, Second Affiliated Hospital, Ministry of Education, Zhejiang University School of Medicine, Key Laboratory of Multiple Organ Failure (Zhejiang University), Hangzhou, China

**Keywords:** microbial pathogen, Asian older adults, global burden of disease, infectious diseases, sociodemographic index

## Abstract

**Background:**

Microbial pathogens–associated infectious diseases significantly threaten older adults, especially in Asia, home to over 60% of the world’s older adults. Age-related immune decline increases infection-related mortality and disability. Using Global Burden of Disease (GBD) 2021 data, we quantify the burden attributable to 34 microbial pathogens in older adults across Asia.

**Method:**

We conducted a cross-sectional analysis of GBD 2021 data to estimate deaths and disability-adjusted life-years (DALYs) attributable to microbial pathogens in individuals aged ≥ 60 years across the Asia continent and 46 of its countries. Microbial pathogens attribution followed the GBD 2021 framework (34 microbial pathogens). Age-standardized death and DALYs rates were calculated, regional variations analyzed using socio-demographic index (SDI), and uncertainty intervals (UIs) estimated via Monte Carlo simulations.

**Findings:**

In 2021, *Streptococcus pneumoniae* (*S. pneumoniae*) was the leading cause of infectious disease burden among the older adults, with 175,929 deaths (95% UI: 151,002–197,658) and 2,799,883 DALYs (95% UI: 2,430,432–3,129,747), followed by *Staphylococcus aureus* (*S. aureus*). By contrast, lower-burden aetiologies included *Listeria monocytogenes*, *Enteropathogenic Escherichia coli*, and *Aeromonas*. The burden varied across regions, with Cambodia having the highest age-standardized DALYs rate (18,186.08 per 100,000), while high-SDI countries like Qatar had lower mortality. *S. pneumoniae* peaked at ages 80–84 years, disproportionately affecting males (17,312 death; 95% UI: 15,215–19,318) compared to females (15,545 death; 95% UI: 12,191–18,471). A strong inverse correlation was observed between SDI and pathogens burden (*p* < 0.01).

**Interpretation:**

Among Asian adults aged ≥ 60 years, the 2021 microbial pathogens–attributable burden was led by *S. pneumoniae*, followed by *S. aureus*. The SDI inverse gradient was strongest for diarrhoeal disease and meningitis, and weaker for lower respiratory infections. Subregional heterogeneity persisted after age-standardization, implying drivers beyond SDI. These findings support prioritizing adult pneumococcal vaccination programs, strengthening hospital infection-prevention and control with a focus on staphylococcal and Klebsiella transmission through antimicrobial stewardship and hand hygiene, and investing in water, sanitation, and hygiene (WASH) infrastructure particularly in community and healthcare settings. Addressing these disparities requires tailored national strategies that align with local pathogens prevalence, health system capacity, and SDI context.

## Introduction

Infectious diseases–caused by bacteria, viruses, parasites, and fungi–remain a critical global health concern, particularly for aging populations (1, 2). As the world’s population continues to age, the global burden of infectious diseases has increased due to the combined complications of age-related immune decline, age-related physiological changes, and multi-morbidity (3). According to the World Health Organization (WHO), the global proportion of individuals aged 60 and over is projected to nearly double, from 12% in 2015 to 22% by 2050 (4).

Asia’s aging population presents distinctive public-health challenges. In 2020, an estimated 414 million people aged ≥65 years lived in Asia—about 54% of the world’s older population—underscoring the region’s centrality to global healthy aging (5, 6). Marked cross-country disparities in infectious-disease burden reflect differences in health-care infrastructure and access, socio-economic conditions, vaccination coverage in older adults, water–sanitation–hygiene, and infection-prevention and control capacity in the context of antimicrobial resistance (AMR) (7, 8). Consequently, many low- and middle-income countries in Asia face greater difficulty meeting the health needs of rapidly aging populations, with higher infection-related morbidity and mortality than in high-income settings (9–11).

This study uses Global Burden of Disease (GBD) 2021 estimates to characterize microbial pathogen–associated infectious disease burden among adults aged ≥ 60 years across the Asia continent and 46 constituent countries. We quantify deaths and disability-adjusted life-years and report age-standardized rates; identify leading and lower-burden pathogens; and examine variation by subregion and Sociodemographic Index to reflect differences in health-system capacity, vaccination coverage, water, sanitation, and hygiene (WASH), and infection-prevention and control. The goal is to provide comparable, decision-relevant evidence to inform targeted vaccination, infection-prevention strategies, and geriatric care across diverse settings in Asia.

## Methods

### Study design

This study utilizes a cross-sectional analysis based on data from the GBD Study 2021 to quantify the burden of 34 microbial pathogens in the older population (aged 60 years and older) across the Asia continent and 46 of its countries (8). The analysis focuses on microbial pathogens–specific death and disability-adjusted life-years (DALYs) attributable to diarrheal diseases, lower respiratory infections (LRIs), and meningitis across various regions of Asia, with the aim of understanding the regional and demographic variations in disease burden.

### Data sources

Data for this study were derived from the GBD 2021 database, which incorporate data from vital registration systems, health surveys, and hospital records. The data also include mortality and morbidity estimates for microbial pathogens from cause-of-death ensemble modeling (CODEm), and vital statistics such as birth rate, population estimates, and socio-demographic indices (SDI) were integrated from multiple global health sources, including the WHO, national health systems, and epidemiological databases. Analyses were conducted for the Asia continent and 46 of its countries, following GBD 2021 geography. In line with GBD 2021 nosology, LRIs in this study refer to non–COVID-19 LRIs and their aetiologies; COVID-19 is modeled as a separate cause and was therefore not pooled with LRI in primary analyses, to prevent double counting and preserve comparability across 1990–2021 (9).

### Study population

The study population includes individuals aged 60 years and older, stratified by sex and region. Population data were disaggregated into age brackets to ensure robust comparisons across age groups, such as 60–64, 65–69, 70–74, 75–79, 80–84, 85–89, 90–94, and 95 + years. Analyses were conducted for the Asia continent aggregate and 46 constituent countries and territories as defined by the GBD 2021 geographical framework. This approach enables examination of regional and national variations in microbial pathogens burden, with particular attention to low- and middle-income settings due to their disproportionately high disease burdens.

### Microbial pathogens selection

Microbial pathogens were prespecified based on clinical relevance and impact in older adults, following the GBD 2021 etiology framework (9–11). Our analysis incorporates the complete set of 34 microbial pathogens modeled in GBD 2021 for the three target infectious syndromes–diarrheal diseases, LRIs, and meningitis–applied to Asia’s population aged ≥ 60 years. These encompass bacterial [e.g., *Streptococcus pneumoniae* (*S. pneumoniae*), *Staphylococcus aureus* (*S. aureus*), *Klebsiella pneumoniae* (*K. pneumoniae*)], viral (e.g., Norovirus, Rotavirus, influenza virus), fungal, and parasitic agents, as detailed in Supplementary Table S1. The selection includes both individually quantified microbial pathogens and methodologically required residual categories to ensure comprehensive syndrome coverage.

To preserve a closed aetiological composition and maintain unbiased microbial pathogens proportions across heterogeneous data, we retained the residual categories as specified in the GBD framework. “Other bacterial pathogen” represents the aggregate burden attributable to bacterial agents not individually modeled due to data sparsity or diagnostic limitations. Similarly, “Other viral aetiologies of LRI” encompasses the viral share of LRIs not accounted for by specifically quantified viruses. Removing these residuals would either prevent etiology shares from summing to the syndrome total (100%) or inflate named organisms through re-normalization (9, 10).

### Outcome measures

The primary outcomes of this study are microbial pathogens–attributable mortality and DALYs. Mortality estimates were calculated based on death rate attributed to each microbial pathogens, while DALYs were computed by combining years of life lost (YLLs) due to premature death and years lived with disability (YLDs) due to infection. Age-standardized rates were calculated to control for differences in population age structure across regions and countries.

### Statistical analysis

Statistical analysis was performed using R software to examine the national and regional variations in microbial pathogens–related mortality and DALYs. Age-standardized death rate (ASDR) and age-standardized DALYs rate were calculated for each microbial pathogens, and regional variations were explored by comparing countries across different SDI levels. Spearman correlation coefficients were used to assess the relationship between SDI and microbial pathogens burden. Locally Weighted Scatterplot Smoothing regression was employed to visualize trends in the data. Monte Carlo simulations were used to generate uncertainty intervals for mortality and DALYs estimates to account for data variability.

## Result

### Asian burden of leading microbial pathogens in 2021

The GBD 2021 database revealed that *S. pneumoniae* was the leading microbial pathogens contributing to the Asian burden of disease, accounting for an estimated 175,929 death (95% UI 151,002-197,658) and 2,799,883 DALYs (95% UI 2,430,432-3,129,747) (Tables 1, 2). It primarily causes pneumonia and meningitis, with concerns over antibiotic and vaccine efficacy. The ASDR was 32.31 per 100,000 population (95% UI 27.56–36.38), and the age-standardized DALYs rate was 480.95 per 100,000 population (95% UI 415.66–538.59). *S. aureus* ranked second, responsible for 162,716 death (95% UI 139,593–182,894) and 2,395,246 DALYs (95% UI 2,085, 884–2,682,101). It is a major cause of healthcare–associated infections and bacteremia, frequently complicated by methicillin resistance. The corresponding ARS of 30.76 death per 100,000 (95% UI 26.15–34.64) and 423.13 DALYs per 100,000 (95% UI 365.89–474.56). *K. pneumoniae* followed, contributing 61,170 death (95% UI 52,618-68,868) and 965,359 DALYs (95% UI 840,746–1,082,919). This pathogen drives hospital-onset pneumonia and sepsis, with high rates of carbapenem resistance in Asia. The ASR of 11.24 death per 100,000 (95% UI 9.60–12.68) and 166.02 DALYs per 100,000 (95% UI 143.85–186.44). In contrast, microbial pathogens such as *Listeria monocytogenes* (foodborne sepsis/meningitis), *Enteropathogenic E. coli* (diarrhoeal disease) and *Aeromonas* were associated with the lowest Asian burdens, contributing the fewest death and DALYs among all estimated pathogens.

**Table 1:** The death counts and age-standardized death rate for specific microbial pathogens across three major infectious syndromes among adults aged ≥ 60 years in Asia, 2021 (both sexes combined).

Characteristic	All 3 infectious syndromes (95% UI)	Diarrheal diseases (95% UI)	Lower respiratory infections (95% UI)	Meningitis (95% UI)
*Streptococcus pneumoniae*				
All-age death counts	175,929 (151,002, 197,658)		172,685 (147,910, 194,261)	3,244 (2,798, 3,762)
Age-standardized death rate	32.31 (27.56, 36.38)		31.76 (27.03, 35.81)	0.55 (0.47, 0.64)
*Staphylococcus aureus*				
All-age death counts	162,716 (139,593, 182,894)		161,625 (138,568, 181,779)	1,090 (924, 1,281)
Age-standardized death rate	30.76 (26.15, 34.64)		30.58 (25.98, 34.45)	0.18 (0.16, 0.22)
Other bacterial pathogen				
All-age death counts	64,131 (53,567, 73,950)		62,418 (51,897, 72,222)	1714 (1,153, 2,476)
Age-standardized death rate	11.84 (9.81, 13.69)		11.55 (9.53, 13.39)	0.29 (0.19, 0.42)
*Klebsiella pneumoniae*				
All-age death counts	61,170 (52,618, 68,868)		59,351 (50,947, 66,923)	1819 (1,474, 2,268)
Age-standardized death rate	11.24 (9.60, 12.68)		10.94 (9.32, 12.35)	0.31 (0.25, 0.38)
Norovirus				
All-age death counts	54,646 (7,480, 115,405)	54,646 (7,480, 115,405)		
Age-standardized death rate	9.81 (1.34, 20.77)	9.81 (1.34, 20.77)		
*Pseudomonas aeruginosa*				
All-age death counts	45,822 (38,870, 51,550)		45,822 (38,870, 51,550)	
Age-standardized death rate	8.67 (7.29, 9.78)		8.67 (7.29, 9.78)	
*Enterotoxigenic E coli*				
All-age death counts	37,163 (17,534, 69,996)	37,163 (17,534, 69,996)		
Age-standardized death rate	6.97 (3.26, 13.16)	6.97 (3.26, 13.16)		
*Escherichia coli*				
All-age death counts	35,274 (30,023, 39,932)		33,624 (28,470, 38,186)	1,650 (1,328, 2027)
Age-standardized death rate	6.53 (5.53, 7.41)		6.25 (5.26, 7.11)	0.28 (0.23, 0.35)
*Campylobacter*				
All-age death counts	34,251 (5,117, 97,138)	34,251 (5,117, 97,138)		
Age-standardized death rate	6.20 (0.92, 17.58)	6.20 (0.92, 17.58)		
*Other viral aetiologies of LRI*				
All-age death counts	34,145 (29,172, 38,368)		34,145 (29,172, 38,368)	
Age-standardized death rate	6.47 (5.47, 7.28)		6.47 (5.47, 7.28)	
Influenza				
All-age death counts	29,576 (17,201, 45,733)		29,576 (17,201, 45,733)	
Age-standardized death rate	5.32 (3.13, 8.17)		5.32 (3.13, 8.17)	
*Acinetobacter baumannii*				
All-age death counts	28,013 (23,154, 33,315)		28,013 (23,154, 33,315)	
Age-standardized death rate	4.97 (4.09, 5.92)		4.97 (4.09, 5.92)	
*Legionella* spp				
All-age death counts	27,647 (23,342, 31,372)		27,647 (23,342, 31,372)	
Age-standardized death rate	5.19 (4.34, 5.90)		5.19 (4.34, 5.90)	
Rotavirus				
All-age death counts	26,763 (14,880, 46,988)	26,763 (14,880, 46,988)		
Age-standardized death rate	4.56 (2.53, 7.98)	4.56 (2.53, 7.98)		
*Cryptosporidium*				
All-age death counts	25,791 (12,587, 50,172)	25,791 (12,587, 50,172)		
Age-standardized death rate	4.92 (2.40, 9.58)	4.92 (2.40, 9.58)		
Adenovirus				
All-age death counts	18,603 (7,956, 37,755)	18,603 (7,956, 37,755)		
Age-standardized death rate	3.36 (1.43, 6.85)	3.36 (1.43, 6.85)		
*Haemophilus influenzae*				
All-age death counts	17,122 (14,669, 19,261)		16,613 (14,200, 18,734)	509 (430, 602)
Age-standardized death rate	3.17 (2.69, 3.58)		3.09 (2.62, 3.49)	0.09 (0.07, 0.10)
*Fungus*				
All-age death counts	16,956 (14,037, 19,919)		16,956 (14,037, 19,919)	
Age-standardized death rate	3.04 (2.50, 3.57)		3.04 (2.50, 3.57)	
*Chlamydia* spp				
All-age death counts	16,265 (13,680, 18,646)		16,265 (13,680, 18,646)	
Age-standardized death rate	2.96 (2.48, 3.39)		2.96 (2.48, 3.39)	
*Group B streptococcus*				
All-age death counts	16,252 (13,798, 18,662)		15,317 (12,933, 17,668)	936 (798, 1,090)
Age-standardized death rate	2.99 (2.52, 3.44)		2.83 (2.37, 3.27)	0.16 (0.13, 0.18)
*Cholera*				
All-age death counts	15,942 (11,628, 20,938)	15,942 (11,628, 20,938)		
Age-standardized death rate	2.65 (1.93, 3.50)	2.65 (1.93, 3.50)		
*Mycoplasma*				
All-age death counts	15,138 (12,916, 17,142)		15,138 (12,916, 17,142)	
Age-standardized death rate	2.79 (2.36, 3.16)		2.79 (2.36, 3.16)	
*Shigella*				
All-age death counts	10,340 (4,943, 19,864)	10,340 (4,943, 19,864)		
Age-standardized death rate	1.80 (0.86, 3.46)	1.80 (0.86, 3.46)		
*Enterobacter* spp				
All-age death counts	10,090 (8,258, 11,826)		10,090 (8,258, 11,826)	
Age-standardized death rate	1.89 (1.53, 2.21)		1.89 (1.53, 2.21)	
*Entamoeba*				
All-age death counts	6,106 (2,340, 13,233)	6,106 (2,340, 13,233)		
Age-standardized death rate	1.05 (0.40, 2.29)	1.05 (0.40, 2.29)		
Non-typhoidal *Salmonella*				
All-age death counts	4,886 (0, 14,997)	4,886 (0, 14,997)		
Age-standardized death rate	0.85 (0.00, 2.60)	0.85 (0.00, 2.60)		
*Polymicrobial*				
All-age death counts	4,425 (2,834, 6,696)		4,425 (2,834, 6,696)	
Age-standardized death rate	0.81 (0.51, 1.23)		0.81 (0.51, 1.23)	
*Clostridium difficile*				
All-age death counts	1926 (1,171, 3,030)			
Age-standardized death rate	0.38 (0.23, 0.59)			
*Viral etiologies of meningitis*				
All-age death counts	1876 (1,610, 2,194)			1876 (1,610, 2,194)
Age-standardized death rate	0.32 (0.27, 0.37)			0.32 (0.27, 0.37)
Respiratory syncytial virus				
All-age death counts	1,558 (934, 2,405)		1,558 (934, 2,405)	
Age-standardized death rate	0.28 (0.17, 0.43)		0.28 (0.17, 0.43)	
*Neisseria meningitidis*				
All-age death counts	1,538 (1,321, 1797)			1,538 (1,321, 1797)
Age-standardized death rate	0.26 (0.22, 0.30)			0.26 (0.22, 0.30)
*Listeria monocytogenes*				
All-age death counts	1,071 (870, 1,297)			1,071 (870, 1,297)
Age-standardized death rate	0.18 (0.15, 0.22)			0.18 (0.15, 0.22)
*Enteropathogenic E coli*				
All-age death counts	338 (107, 723)	338 (107, 723)		
Age-standardized death rate	0.06 (0.02, 0.13)	0.06 (0.02, 0.13)		
*Aeromonas*				
All-age death counts	304 (114, 645)	304 (114, 645)		
Age-standardized death rate	0.06 (0.02, 0.12)	0.06 (0.02, 0.12)		

The residual categories ‘Other bacterial pathogens’ and ‘Other viral aetiologies of LRI’ are retained per GBD 2021 methodological standards to ensure a closed compositional model and to avoid upward bias in estimates for named pathogens. They represent the share of burden attributable to bacterial or viral agents not individually modeled due to data sparsity.

**Table 2:** The DALYs counts and age-standardized death rate for specific microbial pathogens across three major infectious syndromes among adults aged ≥ 60 years in Asia, 2021 (both sexes combined).

Characteristic	All 3 infectious syndromes (95%UI)	Diarrheal diseases (95%UI)	Lower respiratory infections (95%UI)	Meningitis (95%UI)
*Streptococcus pneumoniae*				
All-age DALYs counts	2,799,883 (2,430,432, 3,129,747)		2,732,937 (2,367,197, 3,059,396)	66,946 (58,625, 77,093)
Age-standardized DALYs rate	480.95 (415.66, 538.59)		470.18 (405.51, 527.26)	10.77 (9.41, 12.41)
*Staphylococcus aureus*				
All-age DALYs counts	2,395,246 (2,085,884, 2,682,101)		2,372,318 (2,064,261, 2,658,540)	22,928 (19,761, 26,623)
Age-standardized DALYs rate	423.13 (365.89, 474.56)		419.43 (362.42, 470.77)	3.69 (3.18, 4.29)
Other bacterial pathogen				
All-age DALYs counts	1,000,924 (848,669, 1,149,190)		964,562 (813,414, 1,111,519)	36,362 (25,283, 51,150)
Age-standardized DALYs rate	173.12 (145.93, 199.13)		167.27 (140.26, 193.05)	5.85 (4.04, 8.28)
*Klebsiella pneumoniae*				
All-age DALYs counts	965,359 (840,746, 1,082,919)		928,769 (807,023, 1,044,096)	36,590 (29,817, 45,438)
Age-standardized DALYs rate	166.02 (143.85, 186.44)		160.15 (138.44, 180.20)	5.88 (4.78, 7.31)
Norovirus				
All-age DALYs counts	921,556 (165,336, 1,899,958)	921,556 (165,336, 1,899,958)		
Age-standardized DALYs rate	155.94 (27.64, 322.40)	155.94 (27.64, 322.40)		
*Pseudomonas aeruginosa*				
All-age DALYs counts	681,002 (588,365, 761,853)		681,002 (588,365, 761,853)	
Age-standardized DALYs rate	120.17 (103.01, 134.73)		120.17 (103.01, 134.73)	
*Campylobacter*				
All-age DALYs counts	576,839 (110,165, 1,574,784)	576,839 (110,165, 1,574,784)		
Age-standardized DALYs rate	98.20 (18.50, 268.63)	98.20 (18.50, 268.63)		
*Enterotoxigenic E coli*				
All-age DALYs counts	559,898 (276,326, 1,032,167)	559,898 (276,326, 1,032,167)		
Age-standardized DALYs rate	98.95 (48.39, 183.05)	98.95 (48.39, 183.05)		
*Escherichia coli*				
All-age DALYs counts	546,789 (470,737, 617,514)		514,480 (440,069, 583,489)	32,309 (26,288, 39,395)
Age-standardized DALYs rate	94.74 (81.19, 107.08)		89.48 (76.20, 101.53)	5.26 (4.27, 6.42)
Other viral aetiologies of LRI				
All-age DALYs counts	517,195 (450,569, 577,594)		517,195 (450,569, 577,594)	
Age-standardized DALYs rate	91.21 (78.88, 102.11)		91.21 (78.88, 102.11)	
Rotavirus				
All-age DALYs counts	497,044 (286,609, 869,993)	497,044 (286,609, 869,993)		
Age-standardized DALYs rate	80.84 (46.48, 141.13)	80.84 (46.48, 141.13)		
Influenza				
All-age DALYs counts	477,620 (271,719, 749,730)		477,620 (271,719, 749,730)	
Age-standardized DALYs rate	81.16 (46.67, 126.58)		81.16 (46.67, 126.58)	
*Acinetobacter baumannii*				
All-age DALYs counts	473,728 (394,316, 563,356)		473,728 (394,316, 563,356)	
Age-standardized DALYs rate	79.25 (65.80, 94.25)		79.25 (65.80, 94.25)	
*Legionella* spp				
All-age DALYs counts	426,245 (366,561, 482,019)		426,245 (366,561, 482,019)	
Age-standardized DALYs rate	74.28 (63.39, 84.13)		74.28 (63.39, 84.13)	
*Cryptosporidium*				
All-age DALYs counts	359,770 (177,402, 696,984)	359,770 (177,402, 696,984)		
Age-standardized DALYs rate	64.95 (32.01, 125.84)	64.95 (32.01, 125.84)		
Adenovirus				
All-age DALYs counts	304,502 (134,704, 609,888)	304,502 (134,704, 609,888)		
Age-standardized DALYs rate	51.90 (22.87, 104.16)	51.90 (22.87, 104.16)		
*Cholera*				
All-age DALYs counts	302,666 (222,320, 393,373)	302,666 (222,320, 393,373)		
Age-standardized DALYs rate	48.39 (35.45, 63.11)	48.39 (35.45, 63.11)		
*Fungus*				
All-age DALYs counts	290,979 (243,097, 341,515)		290,979 (243,097, 341,515)	
Age-standardized DALYs rate	48.69 (40.53, 57.16)		48.69 (40.53, 57.16)	
*Haemophilus influenzae*				
All-age DALYs counts	274,839 (239,641, 308,187)		263,332 (228,726, 296,342)	11,507 (9,858, 13,483)
Age-standardized DALYs rate	47.42 (41.10, 53.24)		45.57 (39.35, 51.33)	1.85 (1.58, 2.17)
*Chlamydia* spp				
All-age DALYs counts	266,366 (226,833, 304,314)		266,366 (226,833, 304,314)	
Age-standardized DALYs rate	45.46 (38.59, 51.95)		45.46 (38.59, 51.95)	
*Group B streptococcus*				
All-age DALYs counts	261,303 (225,236, 299,492)		241,188 (206,282, 278,187)	20,115 (17,460, 23,238)
Age-standardized DALYs rate	44.83 (38.42, 51.41)		41.60 (35.38, 47.99)	3.24 (2.80, 3.74)
*Mycoplasma*				
All-age DALYs counts	245,547 (212,895, 277,341)		245,547 (212,895, 277,341)	
Age-standardized DALYs rate	42.14 (36.35, 47.65)		42.14 (36.35, 47.65)	
*Shigella*				
All-age DALYs counts	185,465 (89,493, 354,859)	185,465 (89,493, 354,859)		
Age-standardized DALYs rate	30.61 (14.78, 58.56)	30.61 (14.78, 58.56)		
*Enterobacter* spp				
All-age DALYs counts	154,399 (127,602, 181,022)		154,399 (127,602, 181,022)	
Age-standardized DALYs rate	26.90 (22.13, 31.54)		26.90 (22.13, 31.54)	
*Entamoeba*				
All-age DALYs counts	109,910 (42,512, 235,309)	109,910 (42,512, 235,309)		
Age-standardized DALYs rate	18.04 (6.98, 38.67)	18.04 (6.98, 38.67)		
Non-typhoidal *Salmonella*				
All-age DALYs counts	87,180 (1,113, 263,818)	87,180 (1,113, 263,818)		
Age-standardized DALYs rate	14.37 (0.18, 43.45)	14.37 (0.18, 43.45)		
*Polymicrobial*				
All-age DALYs counts	70,627 (45,726, 105,355)		70,627 (45,726, 105,355)	
Age-standardized DALYs rate	12.08 (7.79, 18.12)		12.08 (7.79, 18.12)	
*Viral etiologies of meningitis*				
All-age DALYs counts	35,706 (31,090, 41,465)			35,706 (31,090, 41,465)
Age-standardized DALYs rate	5.79 (5.03, 6.73)			5.79 (5.03, 6.73)
*Neisseria meningitidis*				
All-age DALYs counts	34,357 (30,003, 39,611)			34,357 (30,003, 39,611)
Age-standardized DALYs rate	5.52 (4.81, 6.37)			5.52 (4.81, 6.37)
*Clostridium difficile*				
All-age DALYs counts	26,668 (16,010, 42,401)			
Age-standardized DALYs rate	4.83 (2.91, 7.66)			
Respiratory syncytial virus				
All-age DALYs counts	26,258 (15,446, 41,021)		26,258 (15,446, 41,021)	
Age-standardized DALYs rate	4.43 (2.63, 6.89)		4.43 (2.63, 6.89)	
*Listeria monocytogenes*				
All-age DALYs counts	20,765 (16,955, 25,070)			20,765 (16,955, 25,070)
Age-standardized DALYs rate	3.38 (2.75, 4.08)			3.38 (2.75, 4.08)
*Enteropathogenic E. coli*				
All-age DALYs counts	5,860 (1996, 12,410)	5,860 (1996, 12,410)		
Age-standardized DALYs rate	0.98 (0.33, 2.07)	0.98 (0.33, 2.07)		
*Aeromonas*				
All-age DALYs counts	4,802 (1868, 10,078)	4,802 (1868, 10,078)		
Age-standardized DALYs rate	0.82 (0.32, 1.73)	0.82 (0.32, 1.73)		

The residual categories ‘Other bacterial pathogen’ and ‘Other viral aetiologies of LRI’ are retained per GBD 2021 methodological standards to ensure a closed compositional model and to avoid upward bias in estimates for named pathogens. They represent the share of burden attributable to bacterial or viral agents not individually modeled due to data sparsity.

DALYs, Disability-Adjusted Life Years.

### National variation in microbial pathogens–related burden

**Figure 1:**
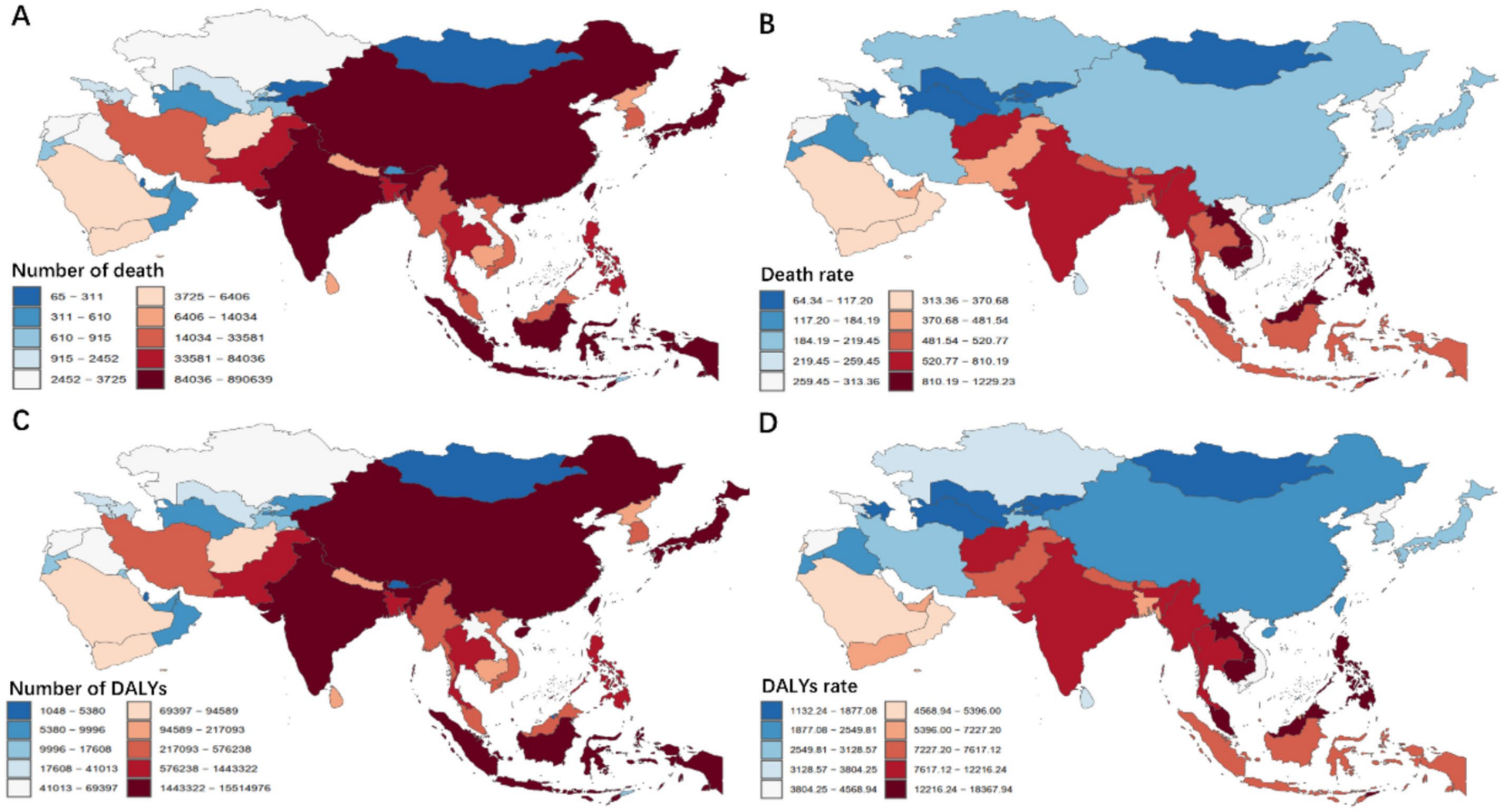
Asian death **(A,B)** and DALYs **(C,D)** patterns attributable to 34 microbial pathogens among the older population for all causes in 2021. DALYs, Disability-Adjusted Life Years.

Significant heterogeneity was observed in the all-pathogens aggregate burden of microbial pathogens–attributable death and DALYs across Asia in 2021 (Figure 1, Supplementary Table S2). India recorded the highest number of death, with an estimated 881,821 death (95% UI: 539,197–1,406,541) (Figure 1A). Indonesia followed, with 89,783 death (95% UI: 52,879–137,731), while Qatar had the lowest, with 65 death (95% UI: 44–94). In terms of ASDR, Cambodia exhibited the highest rate, at 1,217.06 per 100,000 (95% UI: 828.78–1,677.96), followed by Malaysia at 1,091.45 per 100,000 (95% UI: 776.16–1,455.57) (Figure 1B). Conversely, Uzbekistan recorded the lowest ASDR at 71.10 per 100,000 (95% UI: 57.01–86.67). For DALYs, India accounted for the highest burden, with 15,361,362 DALYs (95% UI: 9,691,420–23,971,852), followed by China at 5,008,398 DALYs (95% UI: 3,929,573–6,357,717) (Figure 1C). Qatar reported the lowest DALYs, with 1,187 (95% UI: 792–1,708). After adjusting for population size, Cambodia had the highest age-standardized DALYs rate, at 18,186.08 per 100,000 (95% UI: 12,330.47–25,245.11) (Figure 1D).

**Figure 2:**
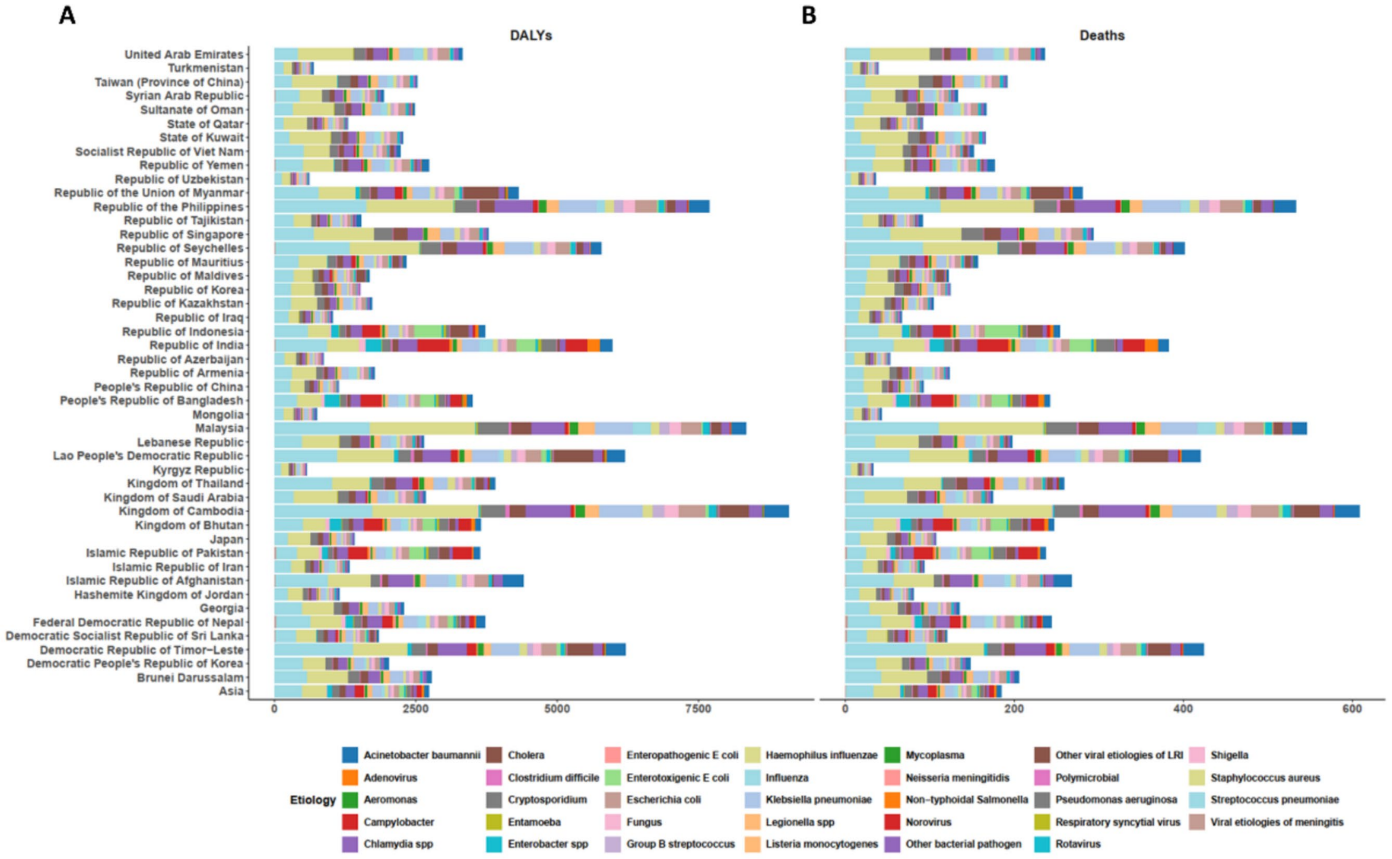
The age-standardized DALYs **(A)** and death **(B)** rate attributable to microbial pathogens across the Asia continent and 46 of its countries for all causes in 2021. DALYs= Disability-Adjusted Life Years.

Across the Asia continent, *S. aureus* was the leading microbial pathogens–attributable cause of death in 26 of its 46 countries (Figure 2B, Supplementary Table S3). Cambodia had the highest ASDR for *S. aureus*, at 129.14 per 100,000 (95% UI: 190.98–173.07), while the Kyrgyz Republic had the lowest ASDR, at 7.62 per 100,000 (95% UI: 6.07–9.41). *S. pneumoniae* was the leading cause of death in 20 regions, with the Philippines reporting the highest ASDR of 111.97 per 100,000 (95% UI: 82.55–133.29). Additionally, *Enterotoxigenic Escherichia coli* was the leading cause of death in Indonesia, with an ASDR of 39.74 per 100,000 (95% UI: 12.22–82.65). Regarding DALYs, *S. aureus* was the leading bacterial cause of DALYs in 25 of 46 countries in Asia (Figure 2A). Cambodia recorded the highest age–standardized DALYs rate for *S. aureus*, at 1878.59 per 100,000 population (95% UI: 1317.32–2541.48), while the Kyrgyz Republic had the lowest rate, at 128.97 per 100,000 (95% UI: 103.22–159.75). *S. pneumoniae* was the leading cause of DALYs in 22 regions, with the Philippines reporting the highest age - standardized DALYs rate of 1618.39 per 100,000 (95% UI: 1220.49–1928.04), and Turkmenistan the lowest, at 155.82 per 100,000 (95% UI: 119.28–198.99).

### Age- and sex-specific burden of specific microbial pathogens in Asia

**Figure 3:**
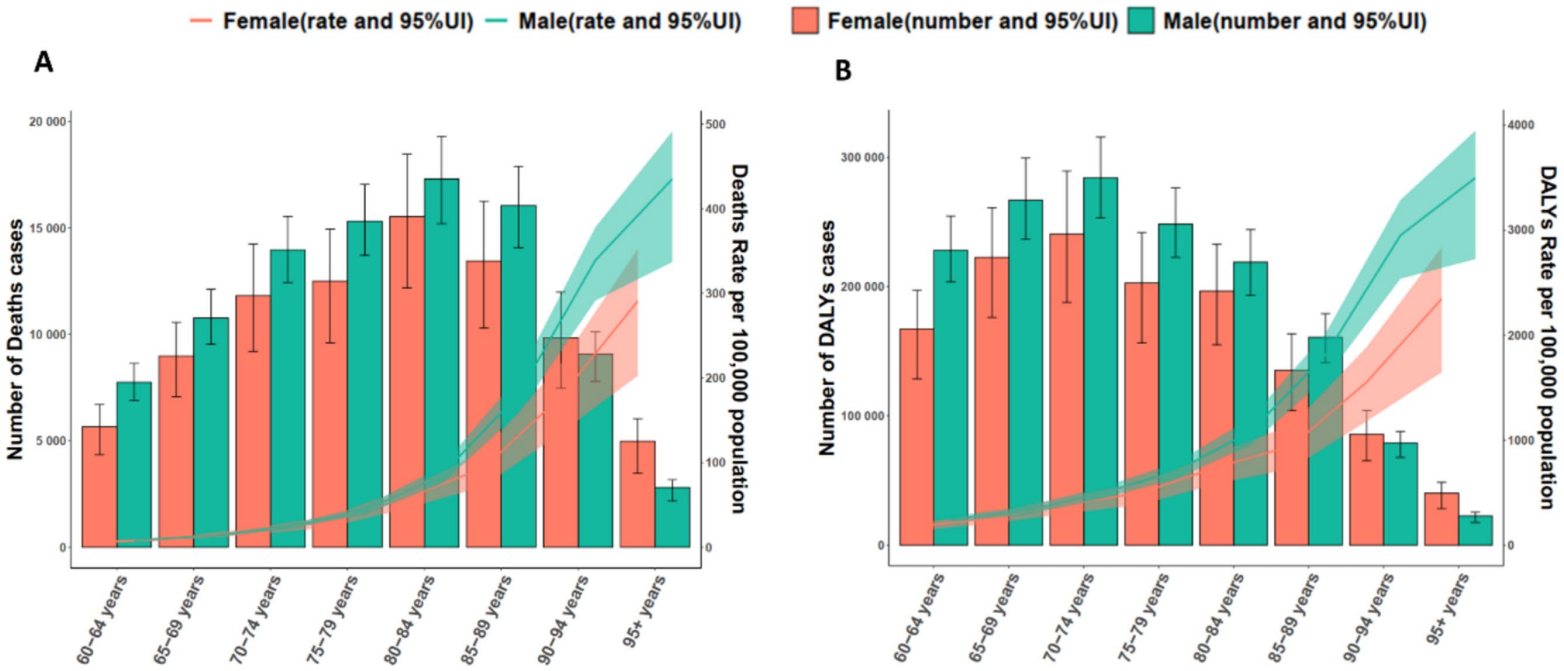
Age- and sex-specific death **(A)** and DALYs **(B)** attributable to all causes by *Streptococcus pneumoniae* in older populations in 2021. DALYs, Disability-Adjusted Life Years.

Across Asia, the burden of *S. pneumoniae* varied markedly by age and sex (Figure 3). The highest burden was observed in those aged 70–74 years in 2021, reaching 284,129 DALYs (95% UI 253,150–315,707) in males and 240,618 (95% UI 187,821–289,478) in females (Figure 3B). The ASR increased substantially in older age groups, with males aged 95 + years experiencing an ASR of 3,496.21 per 100,000 (95% UI: 2,722.82–3,945.15), compared to 2,344.12 per 100,000 (95% UI: 1,646.32–2,834.66) in females. In terms of mortality, *S. pneumoniae* death were highest in the 80–84 years age group, with males having a higher burden (17,312 death; 95% UI: 15,215–19,318) than females (15,545 death; 95% UI: 12,191–18,471) (Figure 3A). The highest ASDR was observed in the 95 + years age group, with an ASDR of 435.10 per 100,000 (95% UI: 337.07–491.31) in males, and 290.79 per 100,000 (95% UI: 202.58–352.36) in females.

**Figure 4:**
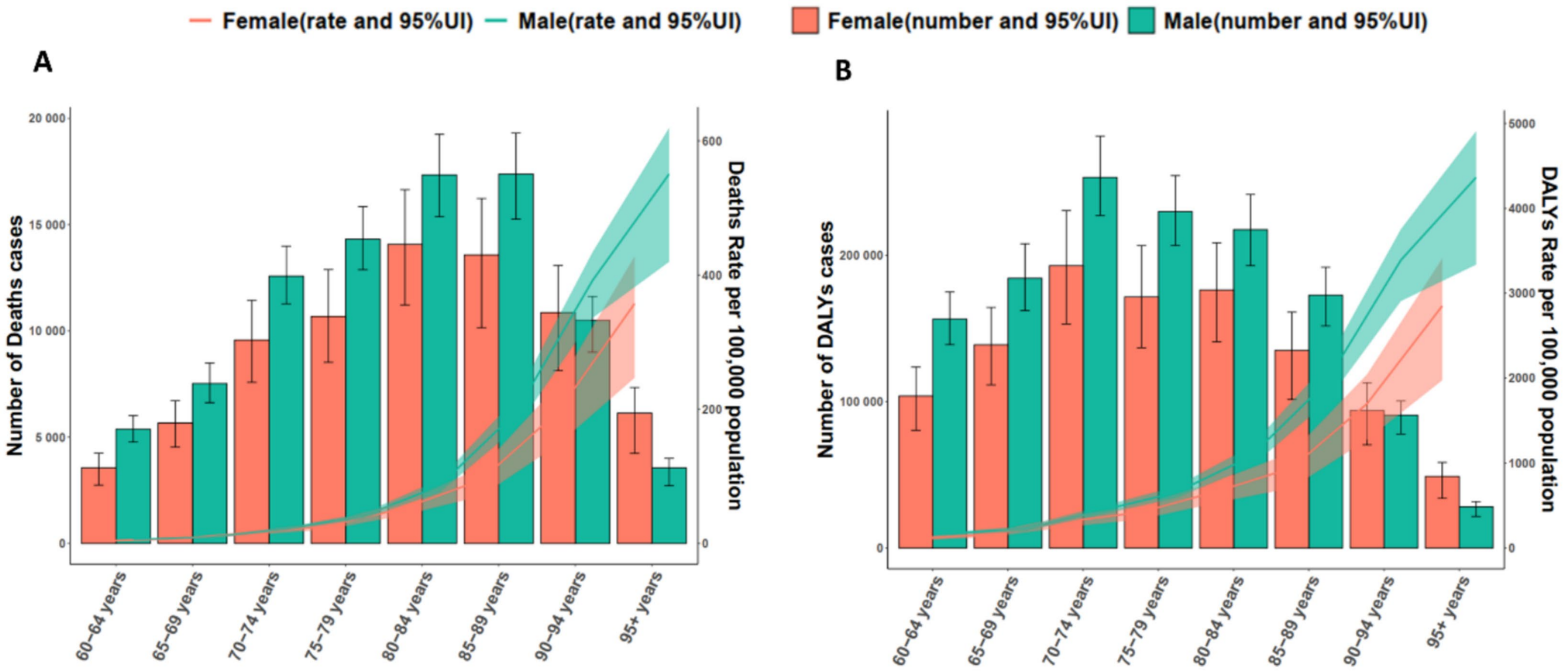
Age- and sex-specific death **(A)** and DALYs **(B)** attributable to all causes by *Streptococcus aureus* in older populations in 2021. DALYs, Disability-Adjusted Life Years.

The burden of *S. aureus* likewise showed clear age- and sex-specific patterns (Figure 4), peaking in 2021 at ages 70–74 years with 253,337 DALYs (95% UI 227,280–281,523) in males and 192,987 (95% UI 153,073–230,672) in females (Figure 4B). The ASR were highest in males aged 95 + years (4,364.81 per 100,000; 95% UI: 3,337.62–4,909.90) and females in the same age group (2,851.19 per 100,000; 95% UI: 1,975.59–3,408.66). Regarding mortality, females in the 80–84 years age group had the highest number of death (14,072 death; 95% UI: 11,221–16,646), while males had a higher burden (17,384 death; 95% UI: 15,258–19,311) in the 85–89 years age group (Figure 4A). For ASDR, males in the 95 + years group exhibited the highest rate at 550.63 per 100,000 (95% UI: 419.80–619.50), while females in the same group had a slightly lower ASDR of 357.57 per 100,000 (95% UI: 247.00–427.75).

### Microbial pathogens–specific disparities across disease categories

**Figure 5:**
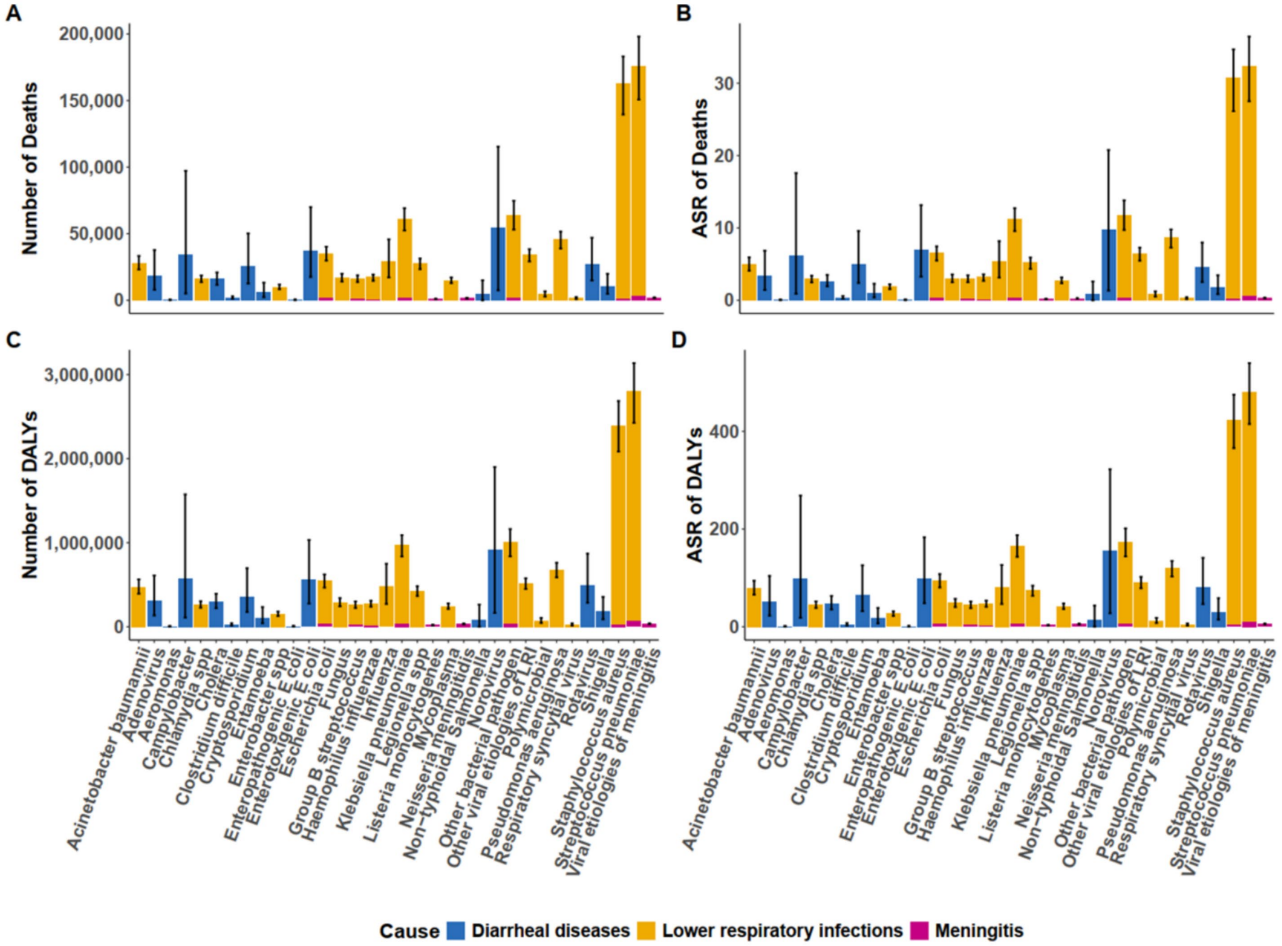
Asian death **(A,B)** and DALYs **(C,D)** attributable to microbial pathogens associated with diarrheal diseases, lower respiratory infections, and meningitis in 2021. DALYs, Disability-Adjusted Life Years.

Across adults aged ≥ 60 years in Asia, *S. pneumoniae* and *S. aureus* were the leading contributors to deaths and DALYs from LRIs in 2021 (Figure 5). *S. pneumoniae* accounted for 172,685 death (95% UI: 147,910–194,261) and 2,732,936.92 DALYs (95% UI: 2,367,196.92–3,059,395.54). *S. aureus* followed closely, contributing 161,625 death (95% UI: 138,568–181,779) and 2,372,318.06 DALYs (95% UI: 2,064,260.70–2,658,540.29). The ASDR for *S. pneumoniae* were the highest, at 31.76 per 100,000 (95% UI: 27.03–35.81), followed by *S. aureus* at 30.58 per 100,000 (95% UI: 25.98–34.45). In terms of DALYs, *S. pneumoniae* had an age-standardized rate of 470.18 per 100,000 (95% UI: 405.51–527.26), while *S. aureus* had 419.43 per 100,000 (95% UI: 362.42–470.77).

For diarrheal diseases in the same age group, *Norovirus* emerged as the leading contributor to mortality, accounting for 54,646 deaths (95% UI: 7,480–115,405), followed by *Enterotoxigenic E. coli*, with 37,163 deaths (95% UI: 17,534–69,996) (Figure 5). *Norovirus* also had the highest age-standardized death rate (ASDR) for diarrheal diseases, at 9.81 per 100,000 (95% UI: 1.34–20.77), with *Enterotoxigenic E. coli* following at 6.97 per 100,000 (95% UI: 3.26–13.16). In terms of DALYs, *Norovirus* had the greatest burden, contributing 921,555.67 DALYs (95% UI: 165,335.70–1,899,958.20), with *Enterotoxigenic E. coli* contributing 559,898.15 DALYs (95% UI: 276,325.72–1,032,166.72).

Regarding meningitis, *S. pneumoniae* and *Neisseria meningitidis* were the primary contributors to mortality and DALYs (Figure 5). *S. pneumoniae* accounted for 3,244 death (95% UI: 2,798–3,762) and 66,946.44 DALYs (95% UI: 58,624.57–77,092.73), while *Neisseria meningitidis* caused 1,538 death (95% UI: 1,321–1,797) and 34,357.48 DALYs (95% UI: 30,003.00–39,610.68). The ASDR for meningitis were highest for *S. pneumoniae* at 0.55 per 100,000 (95% UI: 0.47–0.64), followed by *Neisseria meningitidis* at 0.26 per 100,000 (95% UI: 0.22–0.30).

### Trends and correlations of microbial pathogens burden by socio-demographic index levels

**Figure 6:**
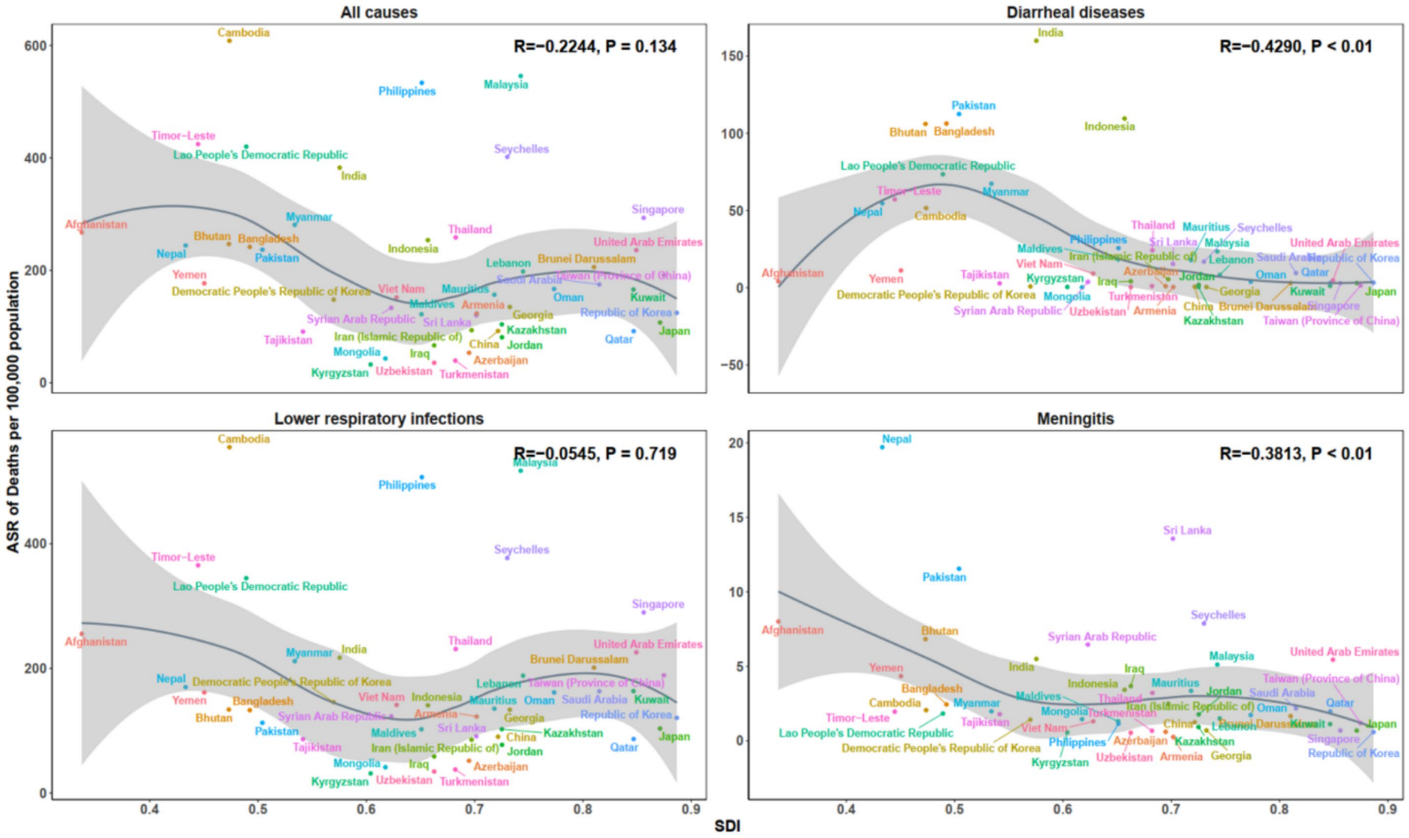
Age-standardized death rate across 46 countries in Asia, by SDI, for all causes, diarrhoeal diseases, lower respiratory infections, and meningitis, 2021. SDI, Socio-demographic Index.

**Figure 7:**
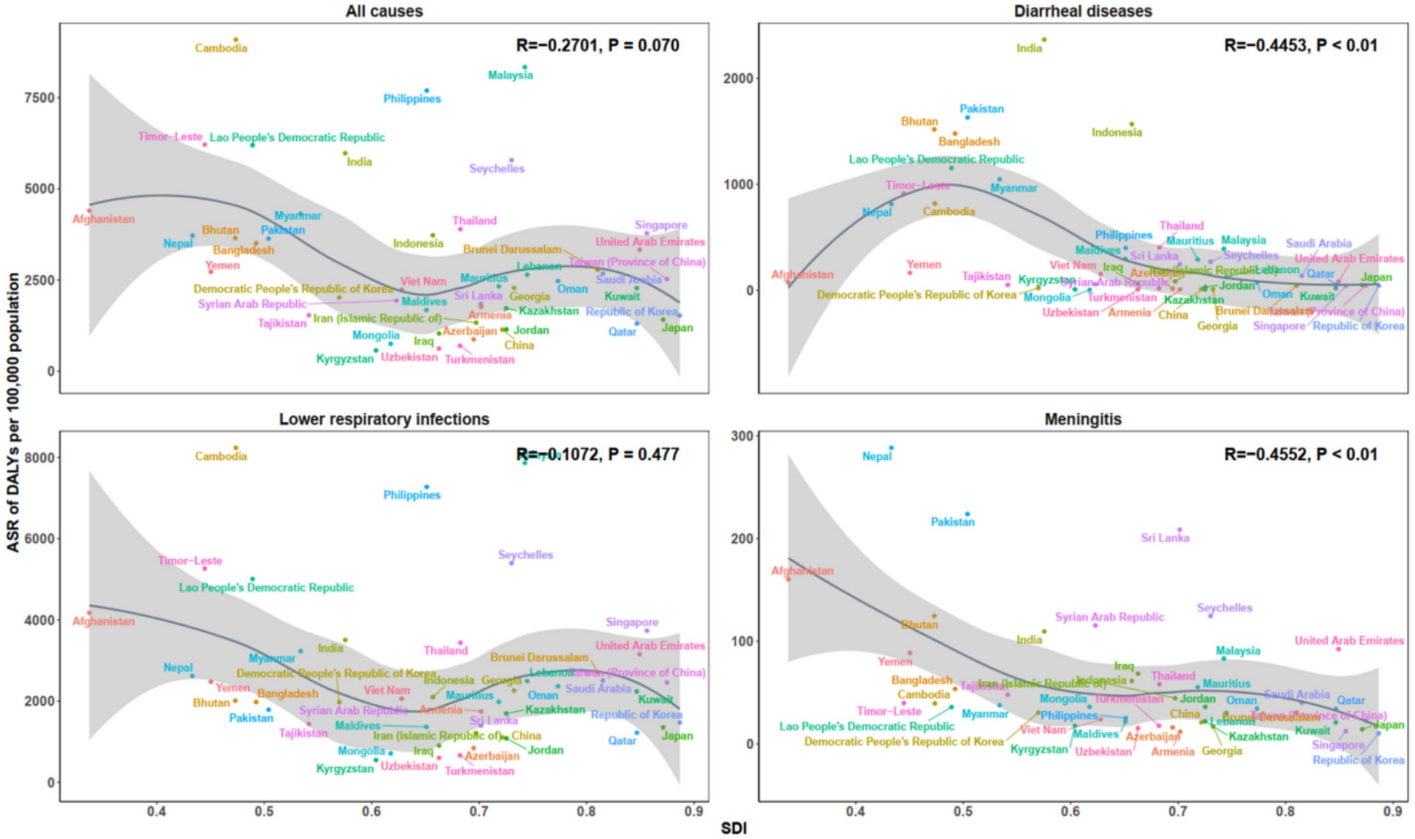
Age-standardized DALYs rate across 46 countries in Asia, by SDI, for all causes, diarrheal diseases, lower respiratory infections, and meningitis in 2021. DALYs, Disability-Adjusted Life Years; SDI, Socio-demographic Index.

At the national level, the ASR of both death and DALYs in 2021 exhibited a strong inverse association with the SDI across all disease categories (Figures 6, 7). A significant negative correlation was observed for all-cause mortality (*R* = −0.224, *p* = 0.134), with higher ASDR found in regions with lower SDI values. A similar trend was observed for all-cause DALYs (*R* = −0.270, *p* < 0.070). This inverse relationship was more pronounced for diarrheal diseases (mortality: *R* = −0.429, *p* < 0.001; DALYs: *R* = −0.445, *p* < 0.001), as well as for meningitis (mortality: *R* = −0.381, *p* < 0.010; DALYs: *R* = −0.455, *p* < 0.010). However, for LRIs, the correlations with SDI were weaker, with mortality (*R* = −0.055, *p* = 0.719) and DALYs (*R* = −0.107, *p* = 0.477) showing no significant association.

## Discussion

This study characterizes microbial pathogen–associated infectious disease burden in adults aged ≥60 years in Asia, using GBD 2021 estimates for the Asia continent and 46 of its countries. We report deaths and disability-adjusted life-years and examine variation by subregion and the SDI; lower respiratory infection estimates refer to non–COVID-19 disease per GBD definitions. The burden is substantial and heterogeneous; within this framework, a small set of microbial pathogens—particularly *S. pneumoniae* and *S. aureus*, with *K. pneumoniae* also contributing—accounts for a large share and shows clear age- and sex-specific differences. These patterns indicate priorities for adult pneumococcal vaccination, strengthened hospital infection-prevention and control in the context of staphylococcal and *Klebsiella* transmission and AMR (12), and investment in WASH and access to care in lower-SDI settings (13, 14).

The findings underscore the substantial burden imposed by *S. pneumoniae*, *S. aureus*, and *K. pneumoniae* among older adults in Asia. *S. pneumoniae*, as the leading microbial pathogens, remains a predominant cause of pneumonia and sepsis, contributing significantly to the burden of LRIs in this population. Its elevated burden is largely attributable to insufficient vaccination coverage among older populations, particularly in low- and middle-income countries (15). Consistent with available country data, this elevated burden coexists with low adult pneumococcal vaccine uptake in several high-burden Asian settings identified in our analysis (e.g., < 2% among Indian adults ≥ 45 years; ~7–9% among older adults in multi-province surveys in China), whereas coverage among seniors is substantially higher in regions with lower *S. pneumoniae* burden, such as the Philippines (~53%) and Japan (≈33–55%, depending on data source) (16–20). These observations are ecological and do not imply individual-level causation, but they align with the cross-country burden patterns observed in our analysis. *S. aureus*, ranking second, presents growing challenges due to the emergence of methicillin-resistant strains, with antibiotic overuse and misuse in healthcare settings across Asia facilitating the spread of resistant variants and complicating treatment approaches (21, 22). *K. pneumoniae*, while less prevalent than the former two microbial pathogens, contributes considerably to mortality, especially among immunocompromised individuals, and its capacity to develop multidrug resistance underscores the urgent need for enhanced infection prevention and antimicrobial stewardship programs in healthcare environments (21, 23).

In contrast, other bacterial pathogens–such as *Listeria monocytogenes*, *Enteropathogenic E. coli*, and *Aeromonas*–are associated with a lower overall burden in Asia. Their limited prevalence in older adults and more focal geographical distribution explain their relatively minor contribution to the disease burden (24, 25). For example, Indonesia reports comparatively higher mortality associated with *Enteropathogenic E. coli*, likely due to persistent challenges in WASH, highlighting the need to strengthen hygiene and sanitation efforts for older adults, who are particularly vulnerable (26). These microbial pathogens therefore remain a threat in settings with poor sanitation and limited health-care access, where outbreaks can have severe consequences (26).

While microbial pathogens–specific burdens reflect underlying biological mechanisms, their population-level impact is strongly shaped by broader structural determinants. We therefore examined how sociodemographic conditions and health-system capacity modulate cross-country disparities in Asia. Across Asian countries, microbial pathogens–attributable mortality and DALYs show an inverse association with the SDI, with the steepest gradients for diarrhoeal diseases and meningitis (8, 27). In lower-SDI settings, shortfalls in WASH, delayed or limited access to care, and underutilization of vaccines contribute to higher mortality and disability (28, 29). In particular, suboptimal coverage of pneumococcal and meningococcal vaccination sustains preventable meningitis burden, underscoring the need to expand adult pneumococcal programs and to adopt context-appropriate meningococcal strategies where indicated (30).

By contrast, LRIs exhibit a weaker and more heterogeneous relationship with SDI, reflecting additional drivers beyond socioeconomic development—multimorbidity in older adults, smoking, ambient air pollution, health-care exposure, and AMR (31, 32). These patterns argue for complementary priorities alongside socioeconomic improvements, including tobacco control and air-quality measures, strengthened infection-prevention and control and antimicrobial stewardship, and investment in geriatric respiratory care.

Marked heterogeneity in microbial pathogens–attributable mortality and DALYs was observed across the region. India and China account for a large share of the regional burden by virtue of population size and uneven access to timely care (24, 25); by contrast, wealthier settings such as Qatar generally achieve lower mortality through earlier diagnosis and access to treatment. Cambodia and Malaysia show elevated age- standardized rates among adults aged ≥60 years—particularly for *S. aureus*—consistent with constrained health-care resources, suboptimal infection-prevention and control, and a higher prevalence of risk factors such as malnutrition (33, 34). Transmission of *S. aureus*, especially in health-care settings, is further amplified in resource-limited environments where capacity for rapid diagnosis, surveillance, and antimicrobial stewardship is restricted (35, 36). The Philippines likewise shows a comparatively high burden of *S. pneumoniae* in our estimates, a pattern plausibly related to challenges in health-care access and vaccination uptake among older adults (37).

Across Asia, age- and sex-specific patterns were evident for the leading microbial pathogens. For *S. pneumoniae*, burden peaked at ages 70–74 and remained high into advanced age; among those ≥95 years, women had higher mortality than men (38), consistent with longer female life expectancy, greater multimorbidity at extreme ages (39, 40), and possible delays in care. For *S. aureus*, DALYs were highest at 70–74 in both sexes, with marked increases in very old males; the prominence of health-care–associated infection and bacteraemia in frail patients underscores the need for strengthened infection prevention and control and early recognition in geriatric care. Accordingly, age- and sex-sensitive strategies include pneumococcal plus seasonal influenza (and, where available, RSV) immunization from age ≥ 70; dysphagia screening and aspiration-prevention with early recognition and post-discharge follow-up after pneumonia (21, 31); enhanced infection prevention and control for staphylococcal burden in very old males (hand hygiene, contact precautions, device-care bundles) coupled with antimicrobial stewardship and rapid diagnostics (27); and, for women ≥ 95 years, targeted outreach to reduce access delays alongside proactive multimorbidity management within geriatric pathways.

These findings further emphasize that diarrhoeal diseases and meningitis contribute materially to the microbial pathogens–attributable burden in older adults across Asia. For diarrhoeal disease, norovirus and *Enterotoxigenic Escherichia coli* are notable contributors: norovirus transmission is often amplified in health-care and long-term care settings (41, 42), whereas *Enterotoxigenic Escherichia coli* is typically linked to contaminated water or food, underscoring the need to strengthen WASH infrastructure—particularly in lower-income settings where sanitation deficits persist (43, 44). For meningitis, *S. pneumoniae* remains the predominant cause of mortality and DALYs among older adults. Although *Neisseria meningitidis* contributes a smaller share overall, it poses meaningful risk where vaccination coverage is suboptimal. These patterns support enhanced pneumococcal and meningococcal immunization strategies, alongside broader preventive measures (45, 46).

Several limitations of this study should be noted. First, the reliance on GBD data, while comprehensive, may not fully capture regional variations in healthcare access and the quality of care, potentially underestimating the burden in certain areas. Second, the study does not account for the full range of factors influencing microbial pathogens transmission and disease outcomes, such as environmental factors, the role of AMR in various regions, or the impact of healthcare system responses. Third, the analysis is based on aggregated national-level data, which may overlook important intra-country disparities in disease burden. Finally, the GBD modeling framework does not explicitly adjust for individual-level comorbidities or AMR patterns, which may lead to underestimation of burden among older adults with multimorbidity or in high-AMR settings.

## Conclusion

This study provides a region-wide assessment of microbial pathogen–associated infectious diseases in adults aged ≥60 years across the Asia continent and 46 of its countries, using GBD 2021 estimates (LRIs defined as non–COVID-19). We quantify deaths and DALYs, showing substantial subregional and SDI heterogeneity and clear age–sex differences. A small set—*S. pneumoniae*, *S. aureus*, and *K. pneumoniae*—accounts for much of the burden, mainly via LRIs. SDI gradients are strongest for diarrhoeal diseases and meningitis, and weaker/more heterogeneous for LRIs. Priorities include adult pneumococcal vaccination and geriatric respiratory care (including seasonal influenza and, where available, RSV immunization); strengthened hospital infection-prevention and control with antimicrobial stewardship (hand hygiene, contact precautions for staphylococcal and Klebsiella transmission, device-care bundles, audit-and-feedback, rapid diagnostics); and investment in WASH and timely access to care in lower-SDI settings, alongside context-appropriate pneumococcal and meningococcal vaccination strategies. Country-specific implementation aligned with local epidemiology and health-system capacity is essential to reduce avoidable mortality and disability.

## Data Availability

The original contributions presented in the study are included in the article/Supplementary material, further inquiries can be directed to the corresponding authors.
