# Supplementary material for: Burden of microbial pathogens–associated infectious diseases in Asian older adults: a systematic analysis derived from the global burden of disease 2021
Source: Front Public Health. 2025 Oct 7;13:1648877. doi: 10.3389/fpubh.2025.1648877 (PMC12537666; doi:10.3389/fpubh.2025.1648877)
Supplement: Supplementary file 4 [file Table_4.DOCX]

**Figure legend**

Fig. 1. Asian death (A/B) and DALYs (C/D) patterns attributable to 34 microbial pathogens among the elderly population for all causes in 2021. Abbreviations: DALYs= Disability-Adjusted Life Years.

Fig. 2. The age-standardized death and DALYs rate attributable to microbial pathogens across the Asia continent and 46 of its countries for all causes in 2021. Abbreviations: DALYs= Disability-Adjusted Life Years.

Fig. 3. Age- and sex-specific death (A) and DALYs (B) attributable to all causes by *Streptococcus pneumoniae* in elder populations in 2021. Abbreviations: DALYs= Disability-Adjusted Life Years.

Fig. 4. Age- and sex-specific death (A) and DALYs (B) attributable to all causes by *Streptococcus aureus* in elder populations in 2021. Abbreviations: DALYs= Disability-Adjusted Life Years.

Fig. 5. Asian death (A/B) and DALYs (C/D) attributable to microbial pathogens associated with diarrheal diseases, lower respiratory infections, and meningitis in 2021. Abbreviations: DALYs= Disability-Adjusted Life Years.

Fig. 6. Age-standardized death rate across 46 countries in Asia, by SDI, for all causes, diarrhoeal diseases, lower respiratory infections, and meningitis, 2021. Abbreviations: SDI = Socio-demographic Index.

Fig. 7. Age-standardized DALYs rate across 46 countries in Asia, by SDI, for all causes, diarrheal diseases, lower respiratory infections, and meningitis in 2021. Abbreviations: DALYs= Disability-Adjusted Life Years; SDI = Socio-demographic Index..


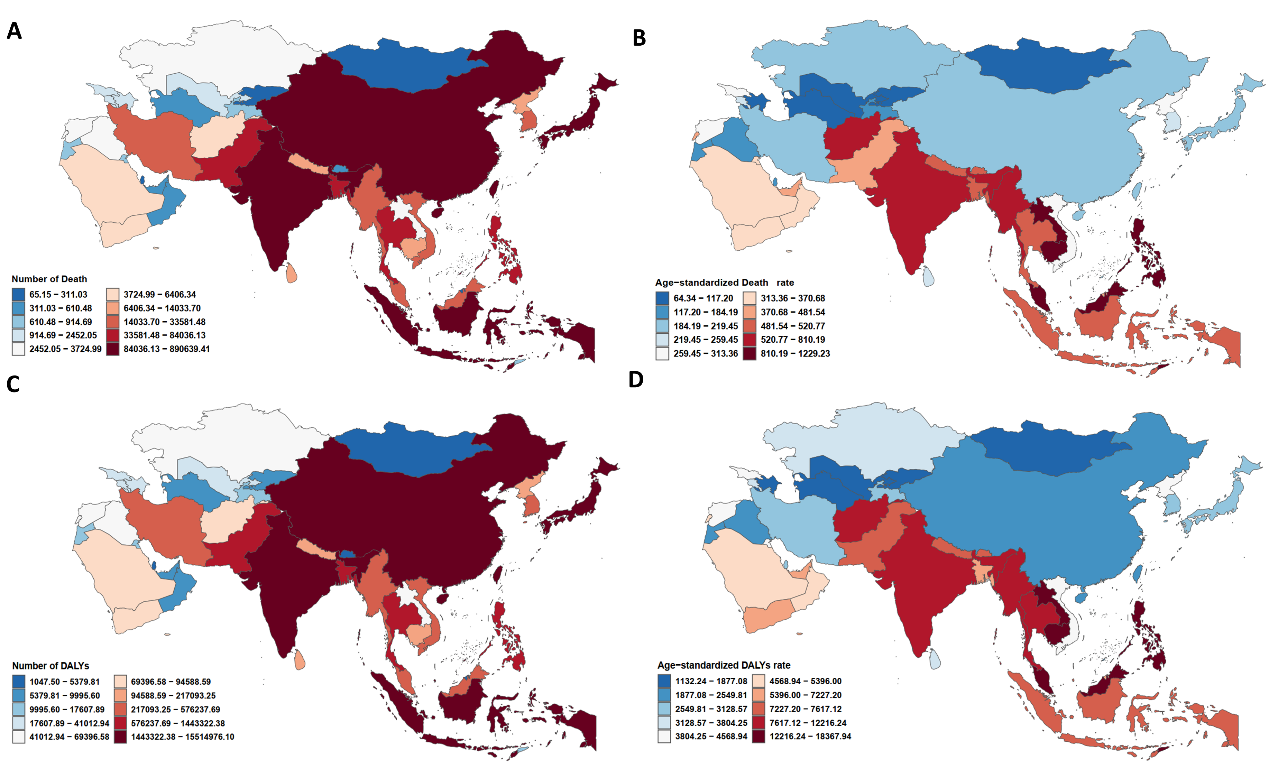


Fig. 1. Asian death (A/B) and DALYs (C/D) patterns attributable to 34 microbial pathogens among the elderly population for all causes in 2021. Abbreviations: DALYs= Disability-Adjusted Life Years.


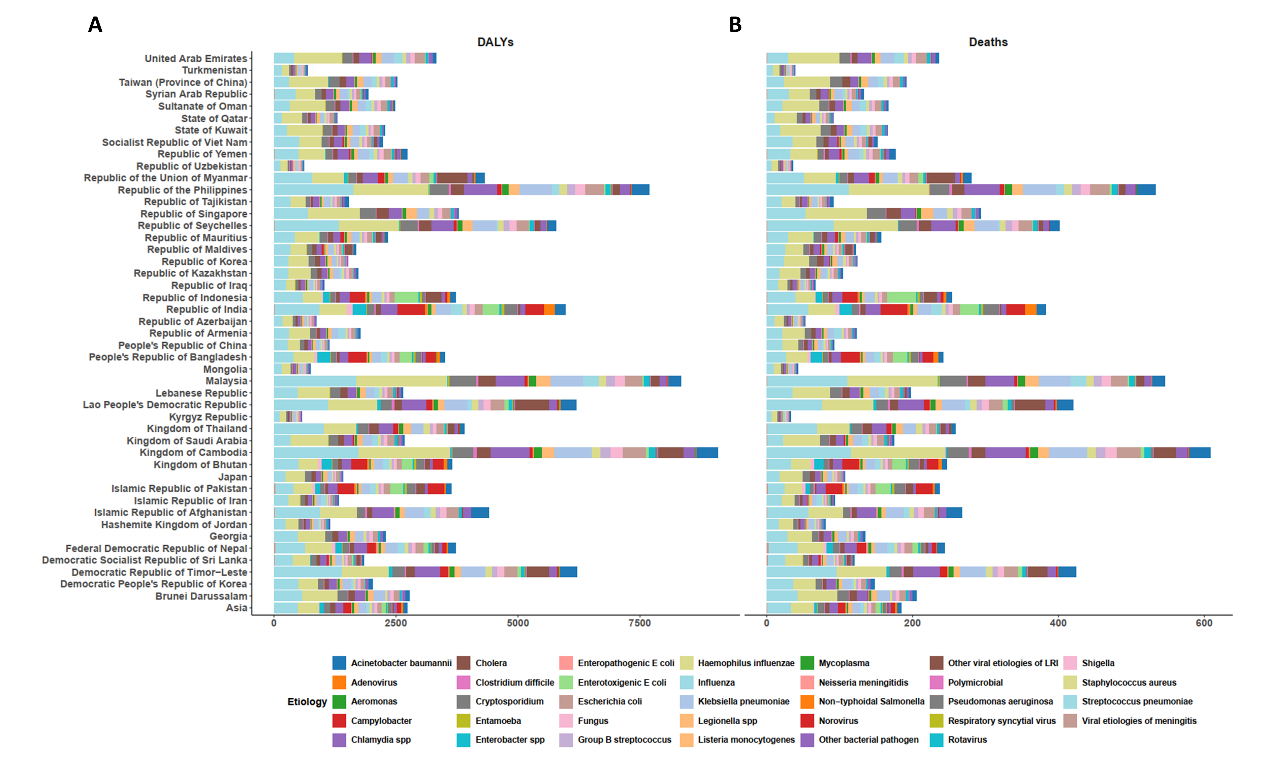


Fig. 2. The age-standardized death and DALYs rate attributable to microbial pathogens across the Asia continent and 46 of its countries for all causes in 2021. Abbreviations: DALYs= Disability-Adjusted Life Years.


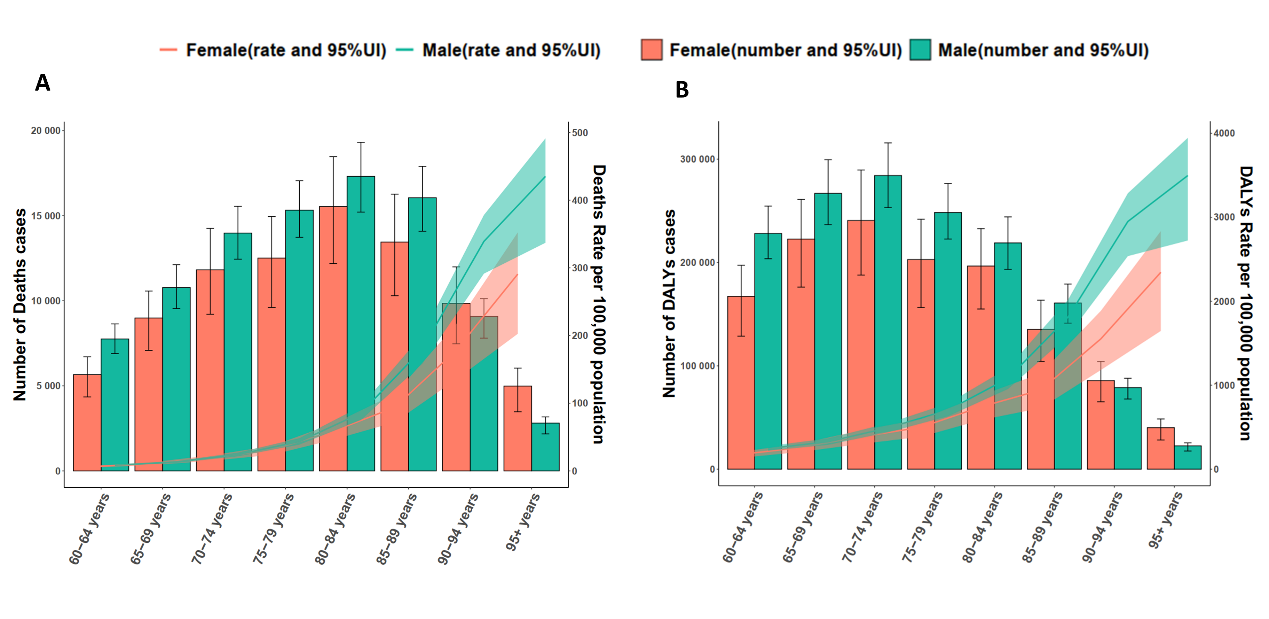


Fig. 3. Age- and sex-specific death (A) and DALYs (B) attributable to all causes by *Streptococcus pneumoniae* in elder populations in 2021. Abbreviations: DALYs= Disability-Adjusted Life Years.


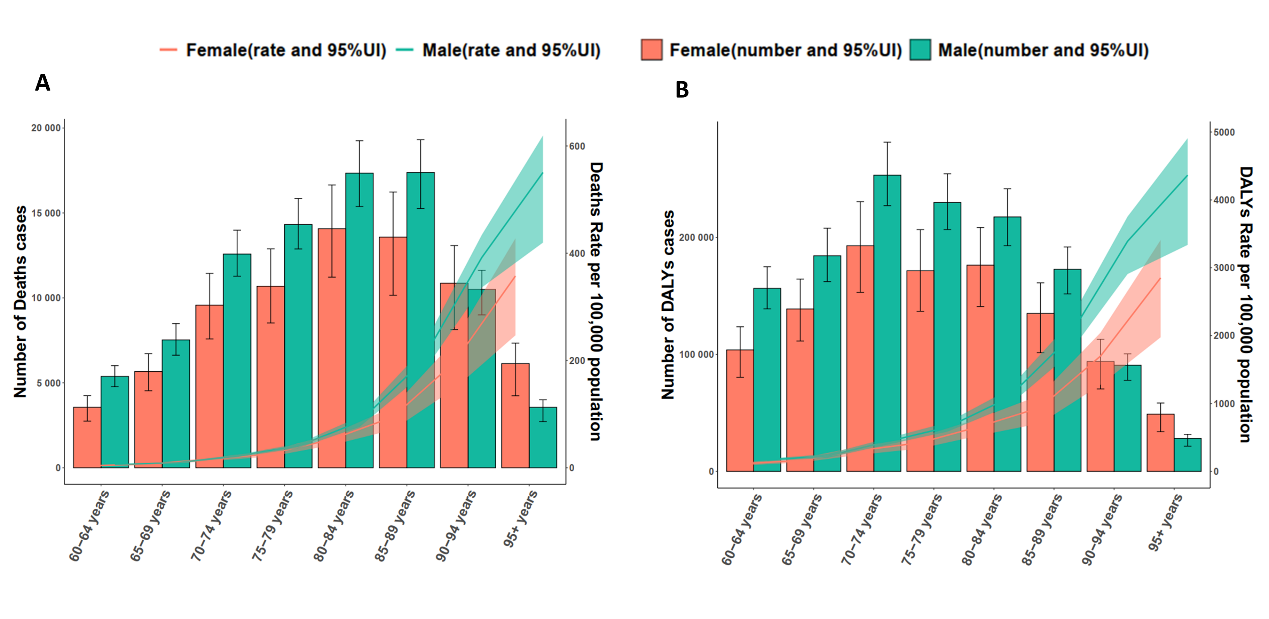


Fig. 4. Age- and sex-specific death (A) and DALYs (B) attributable to all causes by *Streptococcus aureus* in elder populations in 2021. Abbreviations: DALYs= Disability-Adjusted Life Years.


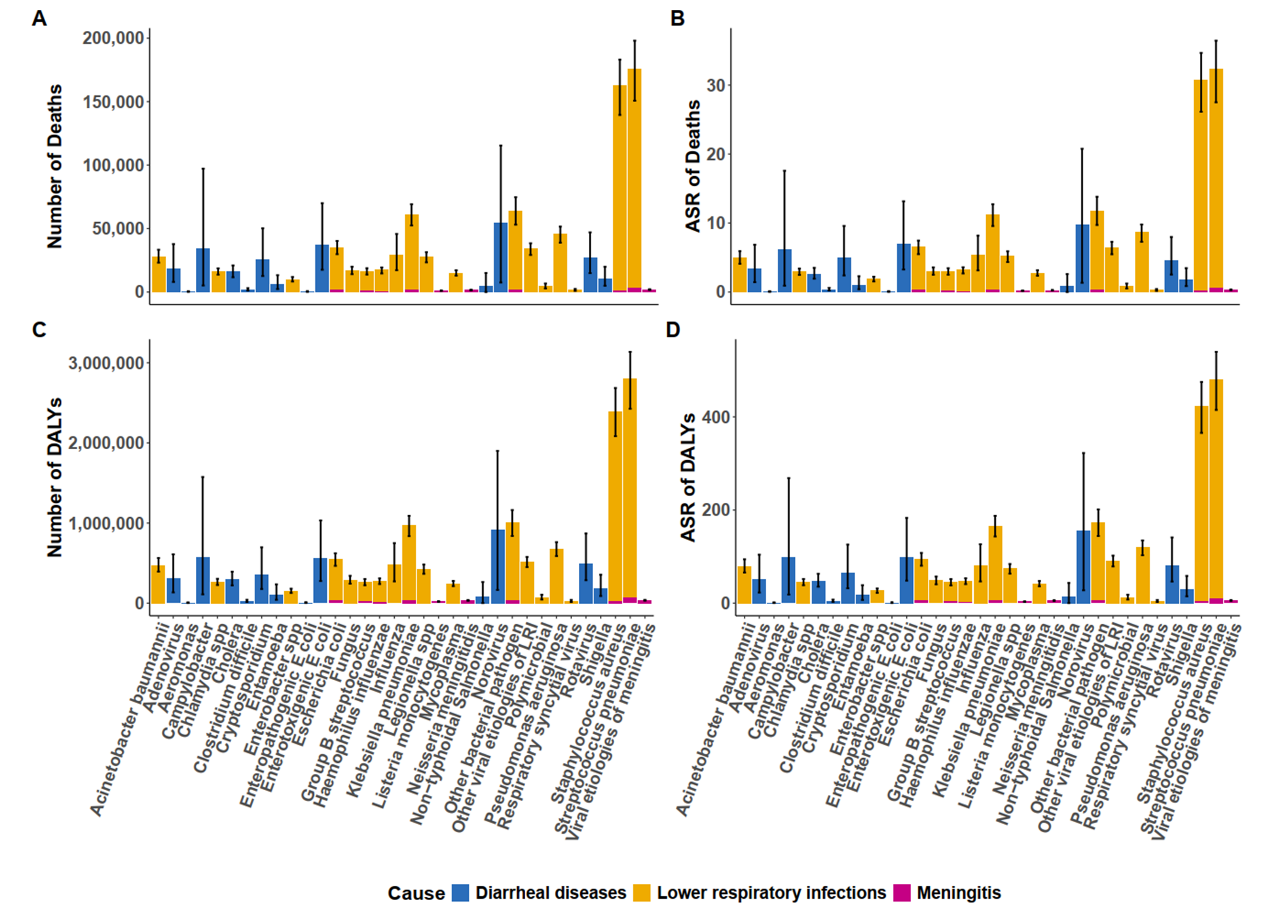


Fig. 5. Asian death (A/B) and DALYs (C/D) attributable to microbial pathogens associated with diarrheal diseases, lower respiratory infections, and meningitis in 2021. Abbreviations: DALYs= Disability-Adjusted Life Years.


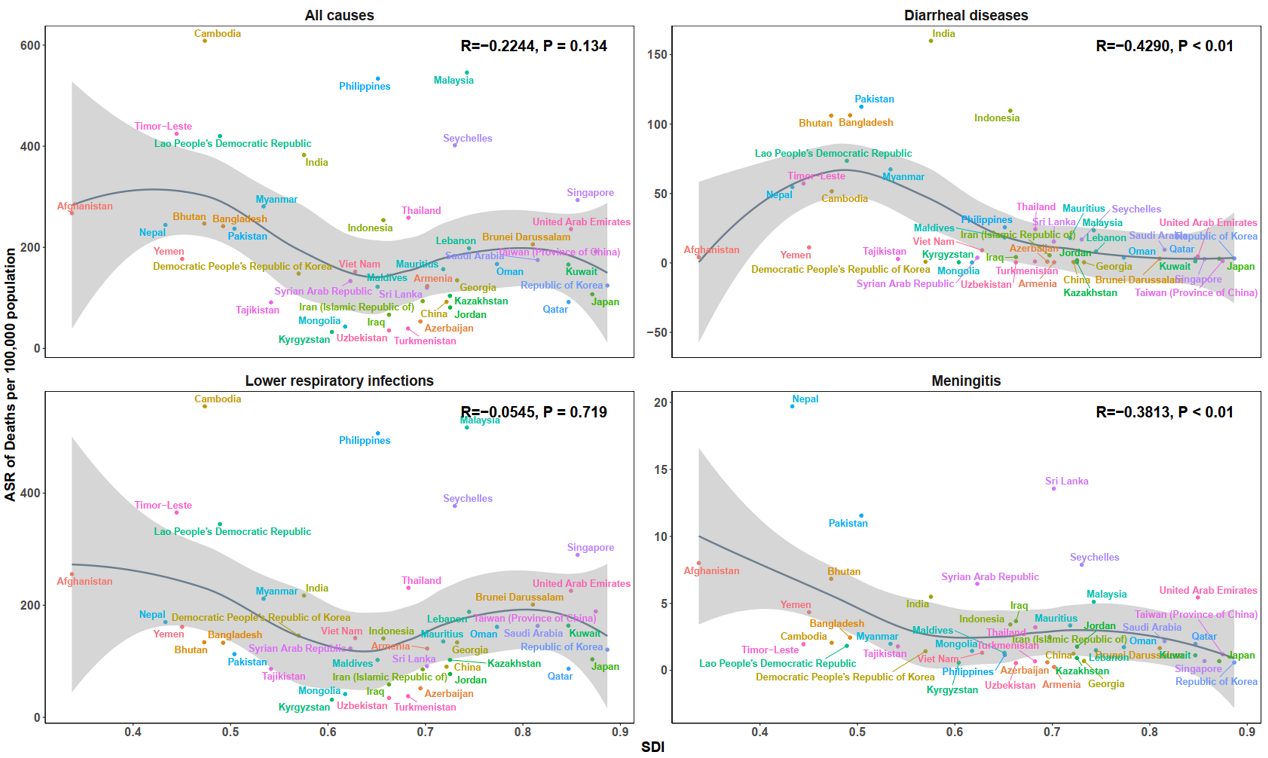


Fig. 6. Age-standardized death rate across 46 countries in Asia, by SDI, for all causes, diarrhoeal diseases, lower respiratory infections, and meningitis, 2021. Abbreviations: SDI = Socio-demographic Index.


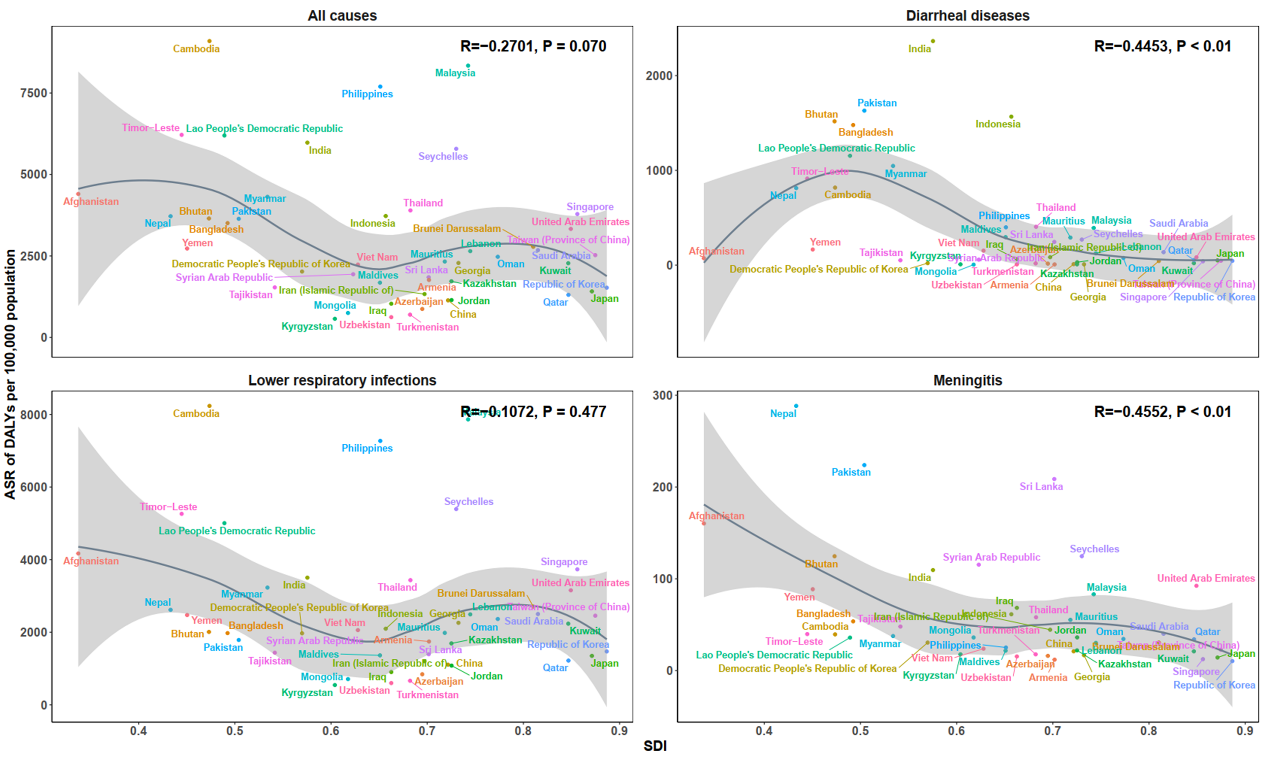


Fig. 7. Age-standardized DALYs rate across 46 countries in Asia, by SDI, for all causes, diarrheal diseases, lower respiratory infections, and meningitis in 2021. Abbreviations: DALYs= Disability-Adjusted Life Years; SDI = Socio-demographic Index.
